# *Pseudomonas aeruginosa* induces tumor pyroptosis and immune activation to enhance checkpoint blockade in colorectal cancer

**DOI:** 10.1007/s00262-025-04266-y

**Published:** 2026-01-03

**Authors:** Yanan Hu, Rong Li, Jiaxi Feng, Ningyi Sun, Wei Zheng, Xin Jin, Guodong Wang, Yi He, Yiwei Hao, Jun Zhang, Ke Ren, Xin Wu

**Affiliations:** 1 The Fourth Affiliated Hospital of Nanjing Medical University, Nanjing, 210032 China; 2 Department of Neurology, Xindu District People’s Hospital of Chengdu, Chengdu, 610500 China; 3 Department of Clinical Microbiology, School of Laboratory Medicine, Chengdu Medical College, Clinical IVD Joint Research Center of Chengdu Medical College-Maccura Biotechnology, Chengdu, 610500 China; 4 Sichuan Provincial Engineering Laboratory for Prevention and Control Technology of Veterinary Drug Residue in Animal-Origin Food, Chengdu Medical College, Chengdu, 610500 China; 5 Taishan Community Health Service Center, Jiangbei New District, Nanjing, 210032 China

**Keywords:** Colorectal cancer, Pyroptosis, *Pseudomonas aeruginosa*, Tumor immune microenvironment, Immune checkpoint blockade

## Abstract

Colorectal cancer (CRC) exhibits limited responsiveness to immune checkpoint inhibitors (ICIs), largely due to its immunosuppressive tumor microenvironment (TME) and poor baseline immunogenicity. Here, we report that *Pseudomonas aeruginosa* (*P. aeruginosa*) triggers caspase-3–dependent pyroptosis in murine CRC MC38 cells, characterized by GSDME cleavage, intracellular reactive oxygen species (ROS) accumulation, and the release of damage-associated molecular patterns (DAMPs). This form of immunogenic cell death promotes robust inflammatory cytokine secretion, upregulation of PD-L1 on tumor cells, and functional maturation of bone marrow–derived dendritic cells (BMDCs) in vitro. In vivo, intratumoral injection of *P. aeruginosa* leads to significant reprogramming of the TME, including increased expression of proinflammatory genes, DC maturation, and enhanced infiltration of CD8^+^ T lymphocytes. Notably, combination therapy with *P. aeruginosa* and an anti-PD-L1 antibody results in synergistic tumor regression, markedly outperforming either monotherapy, without inducing detectable systemic toxicity. Together, our findings reveal that *P. aeruginosa*-induced pyroptosis serves as a potent immunogenic stimulus that reshapes the CRC tumor microenvironment and overcomes resistance to immune checkpoint blockade. This strategy represents a promising approach to enhance immunotherapy efficacy in poorly immunogenic solid tumors.

**Supplementary Information:**

The online version contains supplementary material available at 10.1007/s00262-025-04266-y.

## Introduction

Colorectal cancer (CRC) is the third most commonly diagnosed malignancy and a leading cause of cancer-related mortality worldwide [1, 2]. Despite advancements in surgical techniques, chemotherapy, and targeted therapies, the prognosis for advanced-stage CRC remains poor [3]. In recent years, immunotherapy—particularly immune checkpoint inhibitors (ICIs) targeting the PD-1/PD-L1 axis—has revolutionized cancer treatment [4–6]. However, in CRC, the clinical benefits of ICIs are largely restricted to a small subset of patients with microsatellite instability-high or mismatch repair–deficient tumors [7–10]. The vast majority of CRCs, which are microsatellite stable (MSS), exhibit poor responsiveness to ICI therapy due to an immunologically “cold” tumor microenvironment (TME) characterized by limited immune cell infiltration and weak antigenicity [5, 11]. Therefore, strategies that can convert immune-desert tumors into immunologically active ones are urgently needed.

Induction of immunogenic cell death (ICD) is a promising approach to reshape the TME [12]. Among various forms of ICD, pyroptosis has recently emerged as a potent proinflammatory mode of regulated cell death that can activate innate and adaptive immune responses [13]. Pyroptosis is mediated by pore-forming gasdermin proteins, particularly GSDMD and GSDME, and is typically triggered by inflammatory caspases or caspase-3 [14, 15]. In tumor cells, GSDME cleavage by caspase-3 links classical apoptotic pathways to pyroptosis, resulting in rapid plasma membrane rupture, release of damage-associated molecular patterns (DAMPs), and secretion of proinflammatory cytokines [16]. These events can recruit and activate antigen-presenting cells, promote T cell priming, and ultimately lead to tumor regression [17]. Inducing pyroptosis in tumor cells has thus become an attractive strategy to boost tumor immunogenicity and improve ICI efficacy.

Bacteria-based cancer therapy has garnered increasing interest due to its capacity to selectively colonize tumors, stimulate local immune responses, and serve as delivery platforms for immunostimulatory agents [18, 19]. Among these, *Pseudomonas aeruginosa* is a gram-negative opportunistic pathogen capable of eliciting strong innate immune activation via Toll-like receptor (TLR) signaling and inflammasome pathways [20, 21]. Although *P. aeruginosa* is primarily known for its pathogenicity in immunocompromised hosts, recent studies have suggested that it also possess antitumor potential by inducing inflamed tumor microenvironment [22, 23]. Importantly, in addition to wild-type *P. aeruginosa*, an engineered strain known as *P. aeruginosa*–mannose-sensitive hemagglutinin (PA-MSHA) has been widely studied as an immune modulator in cancer therapy. PA-MSHA expresses mannose-sensitive type I fimbriae, enabling it to potently activate innate immune signaling and promote antigen presentation [22]. Previous studies demonstrated that PA-MSHA can inhibit tumor cell proliferation, sensitize tumors to immune checkpoint inhibitors, and remodel the tumor microenvironment toward an inflamed phenotype [24–26]. These findings underscore the translational potential of *Pseudomonas* strains, either naturally occurring or engineered, to serve as adjuvants that boost anti-tumor immunity. However, the pyroptosis-inducing properties of *P. aeruginosa* in colorectal cancer remain largely unexplored, providing the rationale for our present study.

In this study, we investigated the ability of *P. aeruginosa* to induce GSDME-mediated pyroptosis in murine colorectal cancer cells and examined its downstream effects on the tumor immune microenvironment. We demonstrate that *P. aeruginosa* triggers caspase-3–dependent pyroptotic cell death in MC38 cells, accompanied by increased reactive oxygen species (ROS), loss of mitochondrial function, and release of DAMPs such as HMGB1. This pyroptotic cell death enhances proinflammatory cytokine production, upregulates PD-L1 expression on tumor cells, and promotes the maturation of dendritic cells in vitro. Furthermore, intratumoral administration of *P. aeruginosa *in vivo leads to increased infiltration of CD8^+^ T cells, and overall activation of the TME. Notably, combination therapy with *P. aeruginosa* and anti-PD-L1 antibody achieves superior tumor suppression compared to either treatment alone. These findings provide preclinical evidence supporting the use of bacteria-induced pyroptosis to convert “cold” CRC tumors into immunologically active ones and to enhance the therapeutic efficacy of immune checkpoint blockade.

## Materials and methods

### Cell culture

The murine colorectal cancer cell line MC38 was obtained from Shanghai cell bank and cultured in Dulbecco’s Modified Eagle Medium (DMEM; Gibco), supplemented with 10% fetal bovine serum (FBS; Gibco), 100 U/mL penicillin, and 100 μg/mL streptomycin. Cells were maintained at 37 °C in a humidified incubator with 5% CO_2_ and subcultured every 2–3 days. For bone marrow-derived dendritic cells (BMDCs) generation, bone marrow cells were collected from the femurs and tibias of 6–8-week-old C57BL/6 mice under sterile conditions. Bones were flushed with cold RPMI-1640 medium (Gibco) using a 25G needle, and red blood cells were removed using ACK lysis buffer (Gibco) for 3 min at room temperature. Cells were washed and resuspended in complete culture medium consisting of RPMI-1640 supplemented with 10% fetal bovine serum (FBS, Gibco), 1% penicillin–streptomycin, 2 mM L-glutamine, and 50 μM 2-mercaptoethanol. Cells were seeded at a density of 1 × 10^6^ cells/mL in non-tissue culture-treated Petri dishes (Corning) and incubated at 37 °C in a humidified atmosphere with 5% CO_2_. To induce differentiation into dendritic cells, recombinant murine GM-CSF (20 ng/mL, Beijing Sino Biological, Inc.) and IL-4 (10 ng/mL, Beijing Sino Biological, Inc.) were added to the cultures on day 0. Half of the medium was carefully replaced with fresh cytokine-containing medium on days 3 and 5. On day 6 or 7, loosely adherent and non-adherent cells were harvested as immature BMDCs and used directly or stimulated with different treatments. Cell phenotype was confirmed by flow cytometry.

### Cell viability assay

Cell viability was determined using the Cell Counting Kit-8 (CCK-8, Beyotime). MC38 cells were seeded into 96-well plates at 5 × 10^3^ cells per well and allowed to adhere overnight. Cells were then exposed to *P. aeruginosa* at the indicated concentrations for 48 h. PA-MSHA were provided by Beijing Wante’er Biological Pharmaceutical Co. Ltd. After incubation, 10 μL of CCK-8 reagent was added to each well and incubated at 37 °C for 2 h. Absorbance was measured at 450 nm using a microplate reader (TECAN), and viability was expressed as a percentage relative to the untreated control.

### Transwell invasion assay

Cell invasion was assessed using Transwell inserts (8.0 μm pore size, Corning) precoated with Matrigel (BD Biosciences). MC38 cells (1 × 10^5^) in 200 μL serum-free DMEM were seeded into the upper chambers, and 600 μL of complete medium (10% FBS) was added to the lower chambers. Cells were treated with *P. aeruginosa* at 0, 0.3, 1.0, or 3.0 × 10^7^ CFU/mL. After 24 h, non-invading cells were removed from the upper surface, and invaded cells on the lower surface were fixed with 4% paraformaldehyde and stained with 0.1% crystal violet. Images were acquired using an inverted microscope, and the number of invading cells was quantified in five random fields per insert.

### Wound healing assay

For migration analysis, MC38 cells were seeded in 6-well plates and cultured until confluent. A uniform scratch was made using a sterile 200 μL pipette tip. Cells were washed with PBS and incubated in serum-free DMEM containing PBS or *P. aeruginosa* (0.01, 0.03, or 0.10 × 10^7^ CFU/mL). Images of the scratch area were taken at 0, 12, and 24 h using a phase-contrast microscope (Olympus). The migration rate was calculated using ImageJ software based on the change in wound width over time.

### Live/dead staining assay

MC38 cells were seeded in 24-well plates containing glass coverslips at a density of 1 × 10^5^ cells/well and allowed to adhere overnight. Cells were then treated with *P. aeruginosa* at indicated concentrations (0.3, 1.0, and 3.0 × 10^7^ CFU/mL) for 24 h. Following treatment, cells were washed twice with PBS to remove non-adherent bacteria and then stained with a Calcein-AM/propidium iodide (PI) double staining kit (Beyotime, China) according to the manufacturer’s protocol. Briefly, cells were incubated with 2 μM Calcein-AM and 4.5 μM PI in serum-free medium for 30 min at 37 °C in the dark. After staining, cells were rinsed gently with PBS, and fluorescence images were captured using a fluorescence microscope (Olympus). Live cells exhibited green fluorescence (Calcein-AM), and dead cells were labeled with red fluorescence (PI).

### Flow cytometry for PI staining

To quantify cell death, PI-based flow cytometric analysis was performed. MC38 cells were treated with *P. aeruginosa* (0.3–3.0 × 10^7^ CFU/mL) for 24 h, harvested using trypsin without EDTA, and washed with cold PBS. Cells were then resuspended in PBS containing 5 μg/mL PI (Beyotime, China) and incubated for 10 min at room temperature in the dark. PI-positive (dead) cells were immediately analyzed using a flow cytometer (Cytoflex, Beckman), and data were processed using FlowJo software (v10). At least 10,000 events were recorded per sample.

### Western blot analysis

Following treatment with *P. aeruginosa* (0, 0.1, 0.3, 1.0, and 3.0 × 10^7^ CFU/mL) for 24 h, MC38 cells were lysed in RIPA buffer (Beyotime) supplemented with protease inhibitor cocktail (Selleck). Protein concentrations were determined using the BCA assay (Beyotime). Equal amounts of protein (20–30 μg) were separated by SDS-PAGE and transferred onto PVDF membranes (Millipore). Membranes were blocked with 5% non-fat milk in TBST for 30 min at room temperature and incubated overnight at 4 °C with primary antibodies against full-length caspase-3 (Casp.3-F), cleaved caspase-3 (Casp.3-CL), GSDME, cleaved GSDME (GSDME-CL) and GAPDH (all from Cell Signaling Technology or indicated source). After washing, membranes were incubated with HRP-conjugated secondary antibodies for 1 h at room temperature. Protein bands were visualized using enhanced chemiluminescence (ChemiScope S6) and quantified using ImageJ software.

### Intracellular ROS detection

Intracellular reactive oxygen species (ROS) levels were measured using the fluorescent probe DCFH-DA (Beyotime, China). MC38 cells were seeded in 24-well plates with glass coverslips and treated with *P. aeruginosa* (0.3, 1.0, or 3.0 × 10^7^ CFU/mL) for 24 h. After treatment, cells were washed twice with PBS and incubated with 10 μM DCFH-DA in serum-free medium for 30 min at 37 °C in the dark. Nuclei were counterstained with DAPI (Beyotime) for 5 min. Cells were then rinsed with PBS, and fluorescence images were acquired using a fluorescence microscope (Nikon). For flow cytometric analysis, cells were collected, washed, and resuspended in PBS before being analyzed for DCF fluorescence using a flow cytometer (Cytoflex, Beckman). Data were analyzed with FlowJo software, and ROS levels were expressed as mean fluorescence intensity (MFI) or percentage of DCF-positive cells.

### LDH release assay

Cell membrane integrity was assessed by measuring lactate dehydrogenase (LDH) release using a commercial LDH Cytotoxicity Assay Kit (Beyotime, China). MC38 cells were seeded in 96-well plates and treated with *P. aeruginosa* (0.3, 1.0, or 3.0 × 10^7^ CFU/mL) for 24 h. Supernatants were collected and incubated with LDH reaction mixture according to the manufacturer’s instructions. Absorbance was measured at 490 nm using a microplate reader (TECAN), and LDH release was calculated relative to the PBS control.

### ATP quantification

Intracellular ATP levels were determined using the Enhanced ATP Assay Kit (Beyotime, China). After 24-h treatment with *P. aeruginosa*, MC38 cells were lysed with lysis buffer provided in the kit, and the lysates were centrifuged at 12,000 × g for 5 min. Supernatants were collected, mixed with the luciferase-based ATP detection reagent, and luminescence was measured using a luminometer (Tanon 5260). ATP levels were normalized to the PBS-treated control group.

### HMGB1 ELISA

High mobility group box 1 (HMGB1), IL-6 and TNF-α levels in cell culture supernatants were measured using ELISA kits (Beyotime) following the manufacturer’s protocol. Briefly, supernatants from treated MC38 cells were collected, centrifuged to remove debris, and incubated in ELISA plates pre-coated with anti-HMGB1, IL-6 and TNF-α antibodies. After the addition of biotinylated detection antibody, HRP-conjugated secondary antibody, and substrate solution, absorbance at 450 nm was measured using a microplate reader. HMGB1 concentrations were calculated from standard curves.

### Flow cytometry for PD-L1 expression on tumor cells

MC38 cells were treated with *P. aeruginosa* as described above. After 24 h, cells were harvested, washed with PBS, and stained with anti-mouse PD-L1 antibody (PE-conjugated, clone 10F.9G2, BioLegend) for 30 min at 4 °C in the dark. Cells were then washed and resuspended in staining buffer for flow cytometry analysis. Data were acquired on a CytoFLEX flow cytometer and analyzed using FlowJo software (v10). The percentage of PD-L1–positive cells was quantified relative to the unstained or isotype control group.

### Generation and stimulation of bone marrow–derived dendritic cells (BMDCs)

BMDCs were generated from bone marrow precursors isolated from 6–8-week-old C57BL/6 mice. Briefly, bone marrow cells were flushed from femurs and tibias, filtered, and cultured in RPMI-1640 medium supplemented with 10% FBS, 20 ng/mL GM-CSF, and 10 ng/mL IL-4 (Sino Biological Tech). Fresh cytokines were added every 2 days, and loosely adherent immature BMDCs were harvested on day 6. For stimulation experiments, conditioned medium was collected from MC38 cells treated with *P. aeruginosa* (0.3–3.0 × 10^7^ CFU/mL) for 24 h, filtered through a 0.22 μm membrane, and added to BMDC cultures at a 1:1 ratio with fresh medium. After 24 h of stimulation, BMDCs were harvested and stained for surface markers.

### Flow cytometry for BMDC maturation

After stimulation, BMDCs were collected, washed, and incubated with fluorochrome-conjugated antibodies against CD11c PerCP/Cyanine5.5 (Elabscience, E-AB-F0991J, 1:200), MHC-II PE (Elabscience, E-AB-F0990D, 1:200), CD80 APC (BD Pharmingen™ 560,016, 1:200), and CD86 BV421 (BD Horizon™, 564,198, 1:200) for 30 min at 4 °C. After staining, cells were washed and resuspended in flow buffer. Data were acquired using a flow cytometer (CytoFLEX, Beckman). Within the CD11c^+^ MHC-II^+^ population, CD80^+^CD86^+^ double-positive cells were quantified as mature dendritic cells using FlowJo software.

### Animal experiments and intratumoral injection

Female C57BL/6 mice (6–8 weeks old, weight 18–22 g) were purchased from Chengdu Dossy Experimental Animals Co., Ltd., China, and housed under specific pathogen–free conditions. All animal experiments were approved by the Animal Ethics Committee of Chengdu Medical College (permit no. 2025069). To obtain uniform tumor tissues, MC38 cells (1 × 10^6^) suspended in 100 μL of PBS were inoculated subcutaneously into the right axilla of mice to establish a primary tumor model. When the tumor volume reached about 300 mm^3^, the tumor-bearing the mouse was euthanized and then the tumor tissues were collected. The blood vessels and skin tissue in tumor tissues were removed. After that, non-necrotic tumor tissues were selected to cut into pieces (1 mm^3^) in a sterile Petri dish containing PBS on ice. The right axillary skin of the anesthetized recipient mouse was disinfected and incised. Then, the one piece of tumor tissues was implanted subcutaneously, and the incision was sutured to close the skin. When tumors reached approximately 300 mm^3^ in volume, mice were randomly divided into groups and treated with intratumoral injections of *P. aeruginosa* at different doses of 0.1, 0.3, or 1.0 × 10^7^ CFU in 50 μL PBS for immune analysis. For antitumoral analysis, when tumors reached approximately 80–100 mm^3^ in volume, mice were randomly assigned into four groups (*n* = 5 per group): PBS (control), *P. aeruginosa* alone, anti–PD-L1 antibody (αPD-L1) alone, and combination of *P. aeruginosa* and αPD-L1. *P. aeruginosa* was administered via intratumoral injection at a dose of 1.0 × 10^7^ CFU in 50 μL PBS every three days for a total of three injections. Anti-PD-L1 antibody (Selleck, clone 10F.9G2) was administered intraperitoneally at 20 μg per mouse every five days for three doses. PBS-treated mice received equivalent volumes of vehicle via the same routes. Control mice received PBS injections only. Tumor length (L) and width (W) were measured every three days using digital calipers, and tumor volume was calculated using the formula: volume = (L × W^2^)/2. Mice were also weighed at each time point to monitor potential systemic toxicity. At the study endpoint (day 14 or when control tumors exceeded ethical limits), mice were euthanized, and tumors were excised, photographed, and weighed.

### RNA extraction and qRT-PCR

Tumor tissues were homogenized in TRIzol reagent (Vazyme), and total RNA was extracted following the manufacturer’s protocol. RNA concentration and purity were determined by spectrophotometry (NanoDrop). cDNA was synthesized using a reverse transcription kit (Accurate Biology, China), and quantitative PCR was performed using SYBR Green master mix (Accurate Biology, China) on a real-time PCR system (ABI 7500). Gene expression levels of TNF-α, IFN-γ, and PD-L1 were normalized to GAPDH using the 2⁻ΔΔCt method.

### Flow cytometry for tumor-infiltrating immune cells

Excised tumors were cut into small pieces and digested in RPMI-1640 medium containing 1 mg/mL collagenase IV, 0.1 mg/mL DNase I, and 2.5 U/mL hyaluronidase (Sigma-Aldrich) at 37 °C for 45 min with gentle agitation. Digested tissues were filtered through a 70 μm cell strainer to obtain single-cell suspensions. Cells were washed, counted, and stained with fluorescently labeled antibodies against the following markers: anti-CD45 FITC (Elabscience, E-AB-F1136UC, 1: 200), anti-CD11c PerCP/Cyanine5.5 (Elabscience, E-AB-F0991J, 1:200), anti-MHC-II PE (Elabscience, E-AB-F0990D, 1:200), anti-CD80 APC (BD Pharmingen™ 560,016, 1:200), anti-CD86 BV421 (BD Horizon™, 564,198, 1:200), anti-CD3ε PerCP/Cyanine5.5 (Elabscience, E-AB-F1103UJ, 1:200), anti-CD8α APC (BD Pharmingen™ 553,035, 1:200), and anti-PD-L1 PE (Elabscience, E-AB-F1132D, 1:200). For DC analysis, CD11c^+^MHC-II^+^ cells were gated, and CD80^+^CD86^+^ populations were quantified as mature DCs macrophages. CD3^+^CD8^+^ cells were gated as T cells. PD-L1 expression was analyzed on the CD45^−^ tumor cell population. Flow cytometry was performed using a CytoFLEX, and data were analyzed with FlowJo software (v10).

### Histological and immunohistochemical analysis

Excised tumor tissues were fixed in 4% paraformaldehyde, embedded in paraffin, and sectioned at 4 μm thickness. For histological examination, sections were stained with hematoxylin and eosin (H&E) following standard protocols to assess tumor necrosis and tissue architecture. For proliferation analysis, sections were subjected to Ki67 immunohistochemistry. Briefly, antigen retrieval was performed in citrate buffer (pH 6.0), endogenous peroxidase activity was blocked, and slides were incubated overnight at 4 °C with an anti-Ki67 antibody (Proteintech, 1:200). Detection was performed using HRP-conjugated secondary antibody and DAB substrate (Abcam, ab64261). Slides were counterstained with hematoxylin and imaged under a light microscope.

### Statistical analysis

Data are presented as the mean ± SD. Statistical significance were performed using GraphPad Prism 9.0. Differences among groups were analyzed using one-way analysis of variance (ANOVA) followed by Tukey’s post hoc test. Statistical significance was set at * *P* < 0.05, ** P < 0.01, *** P < 0.001, ns, not significant.

## Results

### *P. aeruginosa* suppresses viability, invasion, and migration of MC38 cells in vitro.

**Fig. 1:**
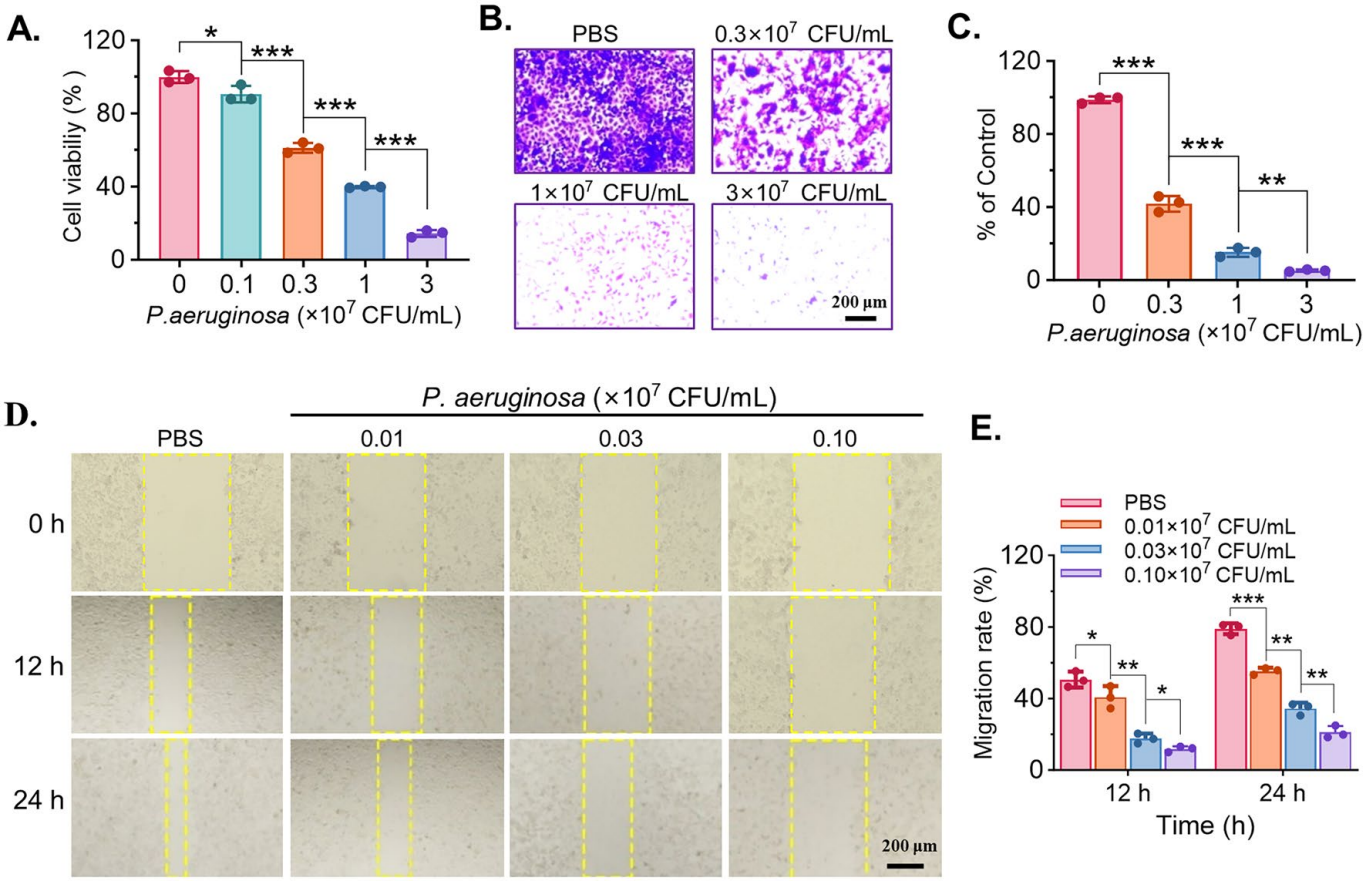
The in vitro antitumoral effects of *Pseudomonas aeruginosa* (*P. aeruginosa*) on murine colorectal cancer of MC38 cells. **A** The cell viabilities of cells treated with increasing concentrations of *P. aeruginosa* (0, 0.1, 0.3, 1, and 3 × 10^7^ CFU/mL) for 24 h, assessed by CCK-8 assay. **B** Representative images of Transwell invasion assay showing cell invasion after treatment with different concentrations of *P. aeruginosa* (PBS, 0.3, 1, and 3 × 10^7^ CFU/mL). Scale bar = 200 µm. **C** Quantification of Transwell experiments, expressed as a percentage of the control (PBS group). **D** Representative images of wound healing assay at 0, 12, and 24 h post-treatment with PBS or *P. aeruginosa* of different concentrations (0.01, 0.03, and 0.10 × 10^7^ CFU/mL). Scale bar = 200 µm. **E** Quantification of migration rates at 12 and 24 h from wound healing assay. Data are presented as mean ± SD (*n* = 3 independent biological replicates). Statistical significance was determined using one-way ANOVA with Tukey’s post hoc test, *P < 0.05, ** P < 0.01, *** P < 0.001, **** P < 0.0001

To evaluate the in vitro antitumor effects of *P. aeruginosa* on colorectal cancer, murine MC38 cells were treated with increasing concentrations of *P. aeruginosa* (0, 0.1, 0.3, 1, and 3 × 10^7^ CFU/mL) for 24 h, and cell viability was assessed using the CCK-8 assay. As shown in Fig. 1A, *P. aeruginosa* significantly reduced the viability of MC38 cells in a dose-dependent manner, with cell viability dropping to less than 20% at the highest concentration (3 × 10^7^ CFU/mL), indicating strong inhibitory effects on cell viability. To further investigate the impact on cell invasion, a Transwell assay was performed. As shown in the representative images (Fig. 1B), the number of invaded cells decreased markedly following exposure to 0.3, 1, and 3 × 10^7^ CFU/mL of *P. aeruginosa*, and quantitative analysis confirmed a significant reduction compared to the PBS control group (Fig. 1C). In parallel, the effects on cell migration were evaluated using a wound healing assay. Representative images captured at 0, 12, and 24 h after treatment revealed a clear delay in wound closure in *P. aeruginosa*-treated groups in comparison to PBS (Fig. 1D). Quantitative analysis demonstrated that both 0.03 and 0.10 × 10^7^ CFU/mL of *P. aeruginosa* significantly inhibited cell migration at 12 and 24 h (Fig. 1E). Taken together, these results demonstrate that *P. aeruginosa* exerts dose-dependent inhibitory effects on colorectal cancer cell viability, invasion, and migration in vitro.

### *P. aeruginosa* induces dose-dependent cell death and caspase-3 activation in MC38 cells

**Fig. 2:**
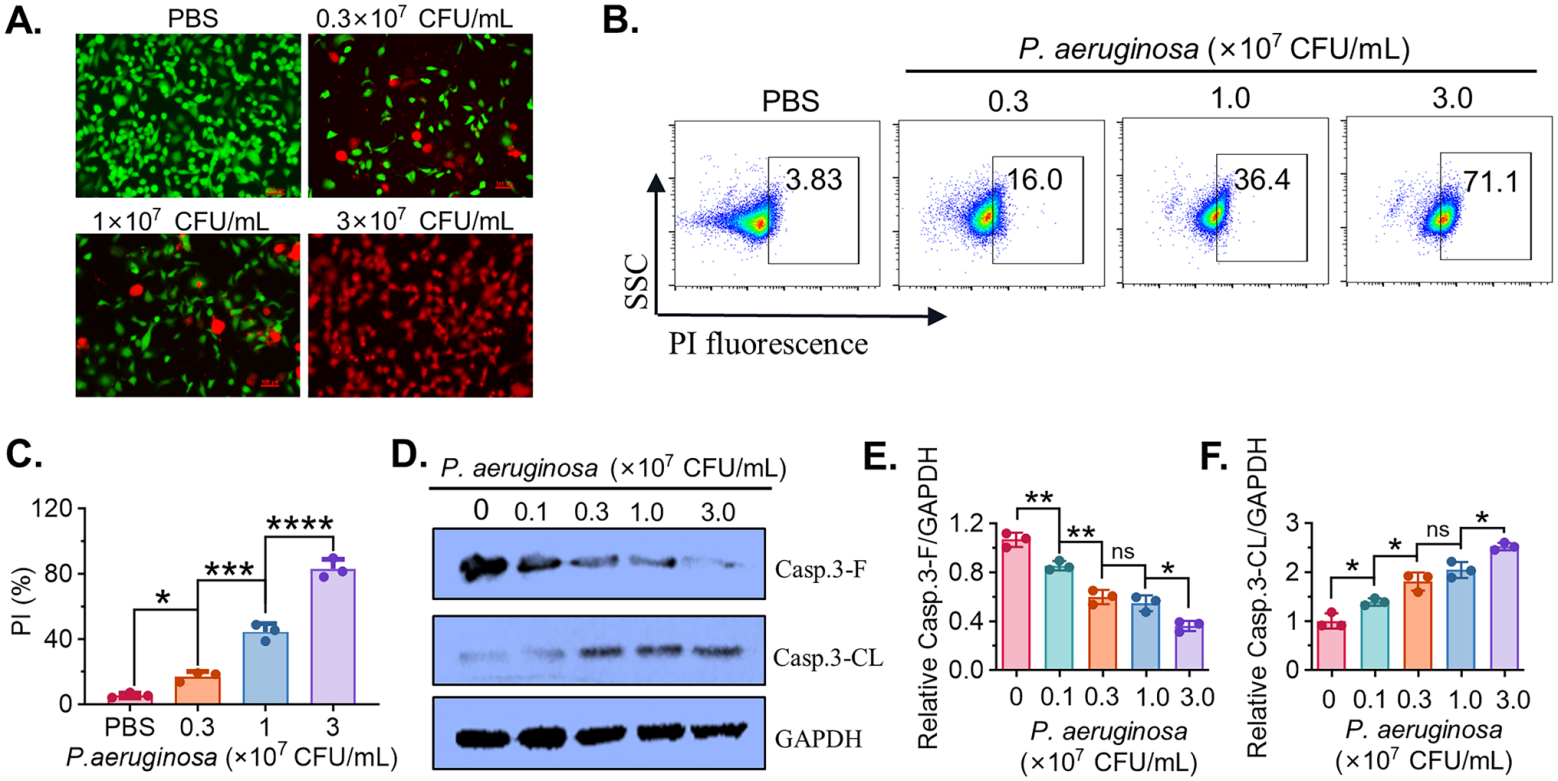
*P. aeruginosa* induces dose-dependent cell death. **A** Live/dead staining of cells treated with increasing concentrations of *P. aeruginosa* (0.3–3 × 10^7^ CFU/mL) for 48 h. Live cells are stained green (Calcein-AM) and dead cells red (PI). Scale bars = 100 μm. **B** Flow cytometry analysis showing the proportion of PI-positive (dead) cells after treatment. **C** Quantification of PI-positive cells of flow cytometric assay, shown as % of total cells. **D** Western blot assay showing expression of full-length (Casp.3-F) and cleaved caspase-3 (Casp.3-CL) after different concentrations of *P. aeruginosa* treatment for 24 h. GAPDH serves as loading control. **E**, **F** Densitometric analysis of Casp.3-F and Casp.3-CL normalized to GAPDH. Data are presented as mean ± SD (*n* = 3 independent biological replicates). Statistical significance was determined using one-way ANOVA with Tukey’s post hoc test, * *P* < 0.05, ** *P* < 0.01, *** *P* < 0.001, **** *P* < 0.0001, ns, not significant

To investigate whether *P. aeruginosa* induces cell death in colorectal cancer cells, MC38 cells were exposed to increasing concentrations of bacteria (0.3, 1.0, and 3.0 × 10^7^ CFU/mL) for 24 h. Live/dead staining showed a gradual increase in red fluorescence (PI-positive dead cells) and a reduction in green fluorescence (Calcein-AM-positive live cells) with increasing bacterial dose, indicating enhanced cell death (Fig. 2A). Flow cytometry analysis further confirmed this observation, revealing a dose-dependent increase in the percentage of PI-positive cells from 3.83% in the PBS group to 71.1% at 3.0 × 10^7^ CFU/mL (Fig. 2B), with statistical quantification showing significant increases at all tested concentrations (Fig. 2C). To explore whether the observed cell death involved apoptotic pathways, the full-length and cleaved caspase-3 was examined by Western blot. Treatment with *P. aeruginosa* led to a reduction in full-length caspase-3 (Casp.3-F) and a corresponding increase in cleaved caspase-3 (Casp.3-CL) in a concentration-dependent manner (Fig. 2D). Densitometric analysis confirmed a significant decrease in Casp.3-Fand a significant elevation of Casp.3-CL (Fig. 2E–F). These findings indicate that *P. aeruginosa* induces cell death in MC38 cells in a dose-dependent manner involving caspase-3 activation.

### *P. aeruginosa* induces pyroptosis for MC38 cells

**Fig. 3:**
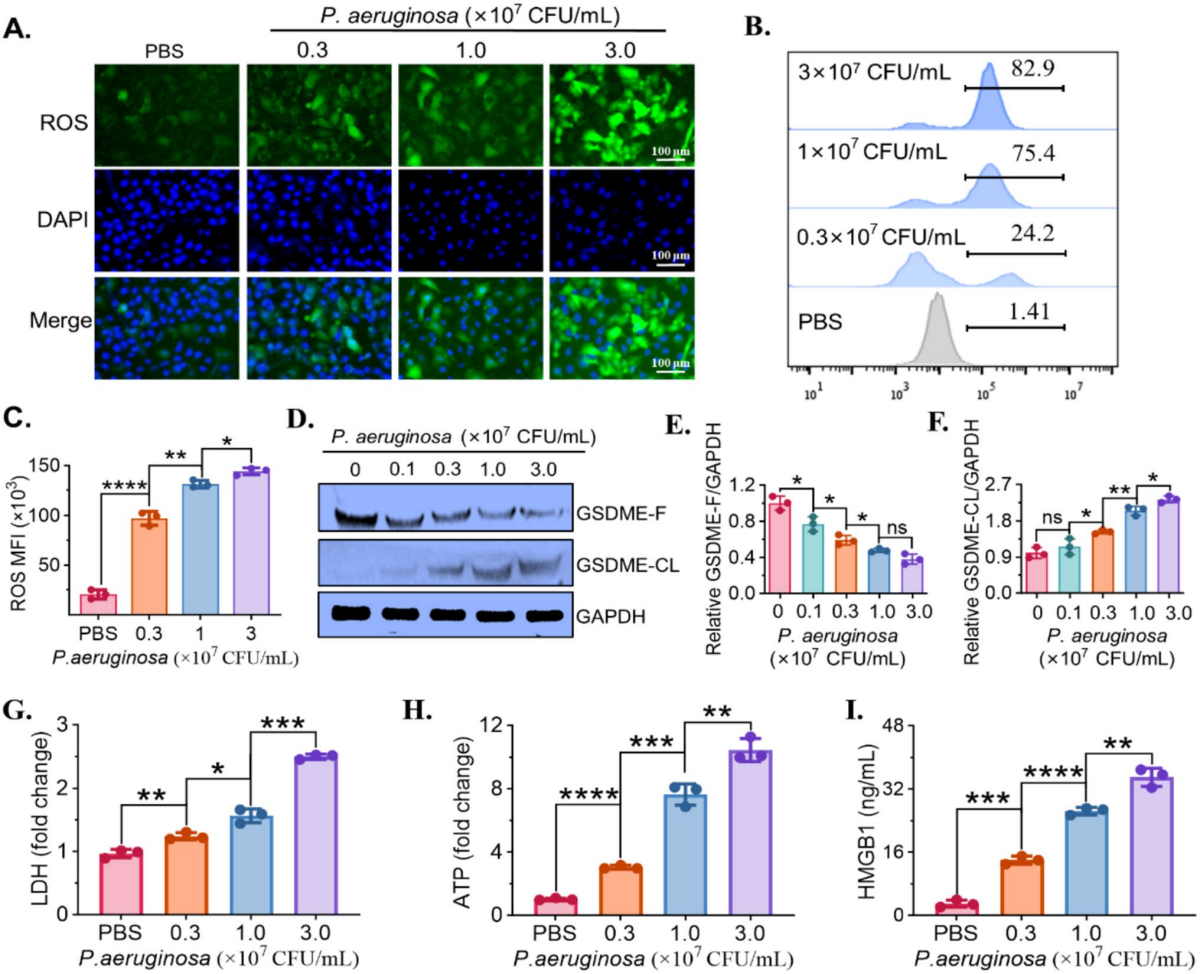
*P. aeruginosa* induces ROS production, GSDME cleavage, and pyroptosis-associated damage. **A** Fluorescence images of ROS levels in MC38 cells treated with *P. aeruginosa* (0.3–3 × 10^7^ CFU/mL) for 24 h, stained with DCFH-DA (green) and DAPI (blue). **B** Flow cytometry analysis of ROS-positive cells. **C** Quantification of ROS mean fluorescence intensity (MFI) for flow cytometry analysis. **D** Western blot of full-length (GSDME-F) and cleaved GSDME (GSDME-CL) after treatment of different concentrations of *P. aeruginosa*. GAPDH was taken as loading control. **E**, **F** Densitometry of GSDME-F and GSDME-CL normalized to GAPDH. **G** Membrane damage determined by lactate dehydrogenase (LDH) release. **H** ATP levels showing mitochondrial dysfunction analyzed by bioluminescence. **I** HMGB1 release measured by ELISA, reflecting pyroptosis-related DAMP release. Data are shown as mean ± SD (*n* = 3 independent biological replicates). Statistical significance was determined using one-way ANOVA with Tukey’s post hoc test, * *P* < 0.05, ** *P* < 0.01, *** *P* < 0.001, **** *P* < 0.0001, ns, not significant

To investigate the involvement of oxidative stress and pyroptosis in *P. aeruginosa*-induced cell death, intracellular ROS levels in MC38 cells were assessed following treatment with increasing concentrations of *P. aeruginosa* (0.3, 1.0, and 3.0 × 10^7^ CFU/mL) for 24 h. Fluorescence staining with DCFH-DA revealed a marked increase in green fluorescence intensity in a dose-dependent manner (Fig. 3A), which was further confirmed by flow cytometry analysis showing a progressive increase in the percentage of ROS-positive cells, from 1.41% in the PBS control to 82.9% at the highest bacterial concentration (Fig. 3B). Quantitative analysis of mean fluorescence intensity (MFI) supported a significant elevation in intracellular ROS levels in response to bacterial exposure (Fig. 3C), suggesting that *P. aeruginosa* triggers oxidative stress in MC38 cells. To determine whether pyroptotic signaling was involved, expression of full-length and cleaved gasdermin E (GSDME-F and GSDME-CL) was examined by Western blot. As shown in Fig. 3D, GSDME-F expression decreased progressively with increased bacterial dose, while cleaved GSDME levels increased accordingly. Densitometric analysis demonstrated a significant reduction in GSDME-F, with a corresponding increase in GSDME-CL expression (Fig. 3E–F), indicating that *P. aeruginosa* induces GSDME-mediated pyroptotic activation. To evaluate membrane integrity, LDH release was measured, and a significant, dose-dependent increase was observed following bacterial treatment (Fig. 3G), reflecting enhanced membrane rupture. Moreover, a significant increase in ATP signals detected in MC38 cell lysates in response to *P. aeruginosa*, particularly at 1.0 and 3.0 × 10^7^ CFU/mL, indicating mitochondrial dysfunction (Fig. 3H). Finally, levels of high mobility group box 1 protein (HMGB1), a damage-associated molecular pattern (DAMP) released during lytic cell death, were significantly elevated in the culture supernatants of *P. aeruginosa*-treated cells (Fig. 3I), supporting the occurrence of pyroptosis-associated damage. These results demonstrate that *P. aeruginosa* induces excessive ROS production, GSDME cleavage, mitochondrial dysfunction, and membrane rupture in MC38 cells, indicative of oxidative stress–driven pyroptotic cell death.

### In vitro immune activation by *P. aeruginosa*-mediated pyroptosis

**Fig. 4:**
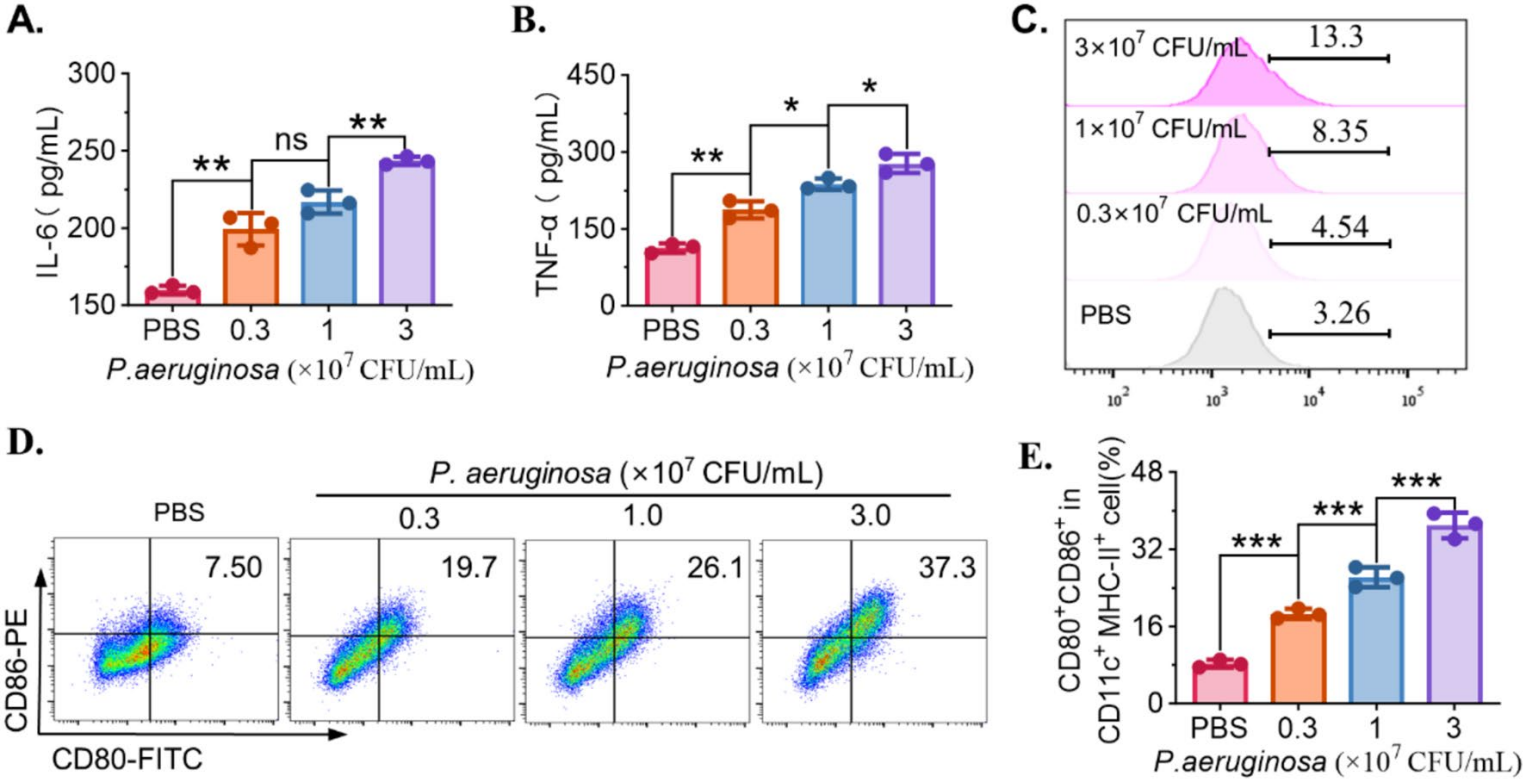
In vitro immune activation mediated by *P. aeruginosa*. **A**, **B** ELISA quantification of IL-6 (**A**) and TNF-α (**B**) secretion from MC38 cells treated with *P. aeruginosa* (0.3–3 × 10^7^ CFU/mL) for 24 h. **C** The expression of PD-L1 on MC38 cells after treatment with different concentrations of *P. aeruginosa*. **D** Representative flow cytometry plots showing the expression of CD80 and CD86 on BMDCs after treatment with cultured medium of *P. aeruginosa*-treated MC38 cells. **E** Quantification of CD80^+^CD86^+^ CD11c^+^ MHC-II^+^-positive for BMDCs. Data are presented as mean ± SD (*n* = 3 independent biological replicates). Statistical significance was determined using one-way ANOVA with Tukey’s post hoc test, * *P* < 0.05, ** *P* < 0.01, *** *P* < 0.001, **** *P* < 0.0001, ns, not significant

To determine whether *P. aeruginosa* modulates the tumor immune microenvironment, inflammatory cytokine secretion was first assessed in MC38 cells treated with increasing concentrations of *P. aeruginosa* (0.3, 1.0, and 3.0 × 10^7^ CFU/mL) for 24 h. ELISA results showed that IL-6 secretion was significantly elevated after treatment of *P. aeruginosa* (Fig. 4A). Similarly, TNF-α levels were increased in a dose-dependent manner (Fig. 4B), indicating that *P. aeruginosa* enhances proinflammatory cytokine production in colorectal cancer cells. In addition, flow cytometry was used to assess PD-L1 expression on MC38 cells following bacterial treatment. As shown in Fig. 4C, PD-L1 expression was progressively upregulated with increasing bacterial concentration, suggesting that *P. aeruginosa* may also modulate immune checkpoint pathways in tumor cells. To evaluate the immunostimulatory potential of *P. aeruginosa*-treated tumor cells, bone marrow–derived cells (BMDCs) were incubated with conditioned media from MC38 cells exposed to different bacterial doses. Flow cytometric analysis demonstrated a dose-dependent increase in the proportion of CD80^+^ CD86^+^ double-positive cells among CD11c^+^ MHC-II^+^ BMDCs (Fig. 4D), and quantitative analysis confirmed significant enhancement of BMDC maturation following treatment with supernatants from *P. aeruginosa*-treated tumor cells (Fig. 4E). These data indicate that *P. aeruginosa* not only promotes inflammatory cytokine secretion and PD-L1 expression in tumor cells, but also enhances the maturation of dendritic cells, thereby potentially contributing to immune activation within the tumor microenvironment.

### The immune regulation of *P. aeruginosa* on the tumor microenvironments

**Fig. 5:**
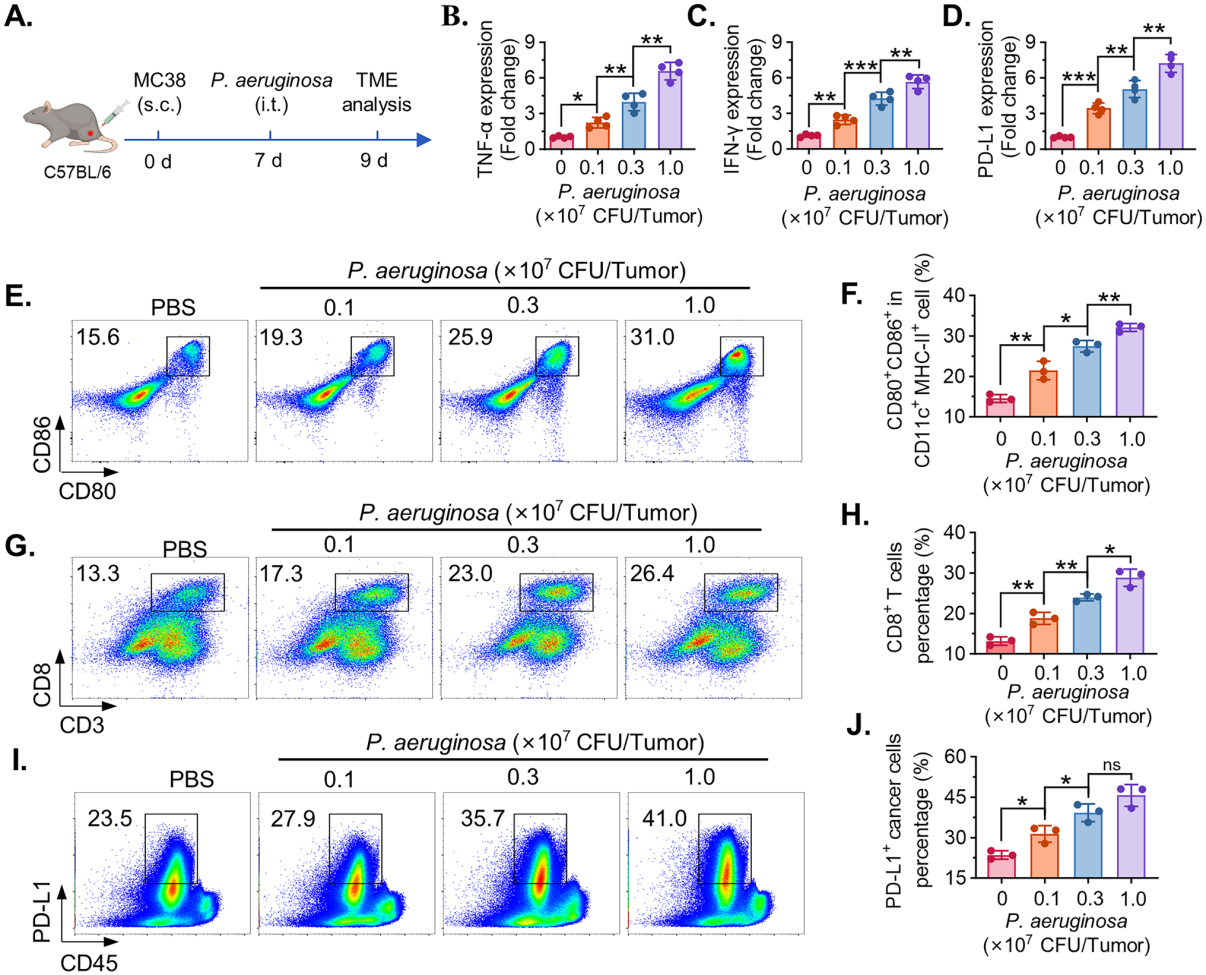
In vivo immune modulation by *P. aeruginosa* affects on MC38 tumor tissues. **A** The schematic treatment timeline. **B–D** qRT-PCR analysis of proinflammatory and immune regulatory genes in tumors tissues after intratumoral injection of *P. aeruginosa* (0.1–1.0 × 10^7^ CFU/tumor), including TNF-α (B), IFN-γ (C), and PD-L1 (D) (*n* = 4 mice per group). **E** Representative flow cytometry plots showing CD80 and CD86 expression on dendritic cells (CD11c^+^ MHC-II^+^ DCs). **F** Quantification of CD11c^+^ MHC-II^+^ CD80^+^ CD86^+^ DCs. **G**, **H** Flow cytometry analysis of CD3^+^ CD8^+^ tumor-infiltrating T cells. **I**, **J** Flow cytometry plots showing PD-L1 expression on CD45^−^ tumor cell populations (*n* = 3 mice per group). Data are presented as mean ± SD. Statistical significance was determined using one-way ANOVA with Tukey’s post hoc test. * *P* < 0.05, ** *P* < 0.01, *** *P* < 0.001, **** *P* < 0.0001, ns, not significant

To investigate the impact of *P. aeruginosa* on the tumor immune microenvironment in vivo, MC38 tumor–bearing mice were intratumorally injected with *P. aeruginosa* at doses ranging from 0.1 to 1.0 × 10^7^ CFU/tumor, and tumor tissues were harvested for analysis. (Fig. 5A) Quantitative RT-PCR revealed a dose-dependent increase in the expression of proinflammatory cytokines and immune modulatory genes within tumors. Specifically, *P. aeruginosa* treatment significantly elevated TNF-α (Fig. 5B) and IFN-γ (Fig. 5C) mRNA levels, suggesting enhanced inflammatory signaling, while PD-L1 expression was also significantly upregulated by the treatment of *P. aeruginosa* (Fig. 5D), indicating potential immune checkpoint activation. To further characterize changes in immune cell phenotypes, flow cytometry was performed to assess dendritic cell maturation. As shown in Fig. 5E, the proportion of CD80^+^CD86^+^ cells among CD11c^+ ^MHC-II^+^ dendritic cells (DCs) increased in a dose-dependent manner following bacterial treatment, and quantification confirmed a significant enrichment of mature DCs (Fig. 5F). Analysis of tumor-infiltrating lymphocytes revealed that the proportion of CD3^+^CD8^+^ T cells was significantly elevated in tumors treated with *P. aeruginosa* compared to PBS controls (Fig. 5G–H), reflecting enhanced cytotoxic T cell infiltration. Furthermore, flow cytometry analysis of tumor cells (CD45^−^ population) showed increased PD-L1 expression in response to bacterial treatment (Fig. 5I), and statistical quantification demonstrated a significant elevation of PD-L1^+^ tumor cells in a dose-dependent manner (Fig. 5J). These results indicate that intratumoral administration of *P. aeruginosa* promotes immune activation in the tumor microenvironment by enhancing proinflammatory gene expression, inducing maturation of DCs, increasing CD8^+^ T cell infiltration, and upregulating PD-L1 expression on tumor cells.

### In vivo combined antitumor activity of *P. aeruginosa* and immune checkpoint inhibitor

**Fig. 6:**
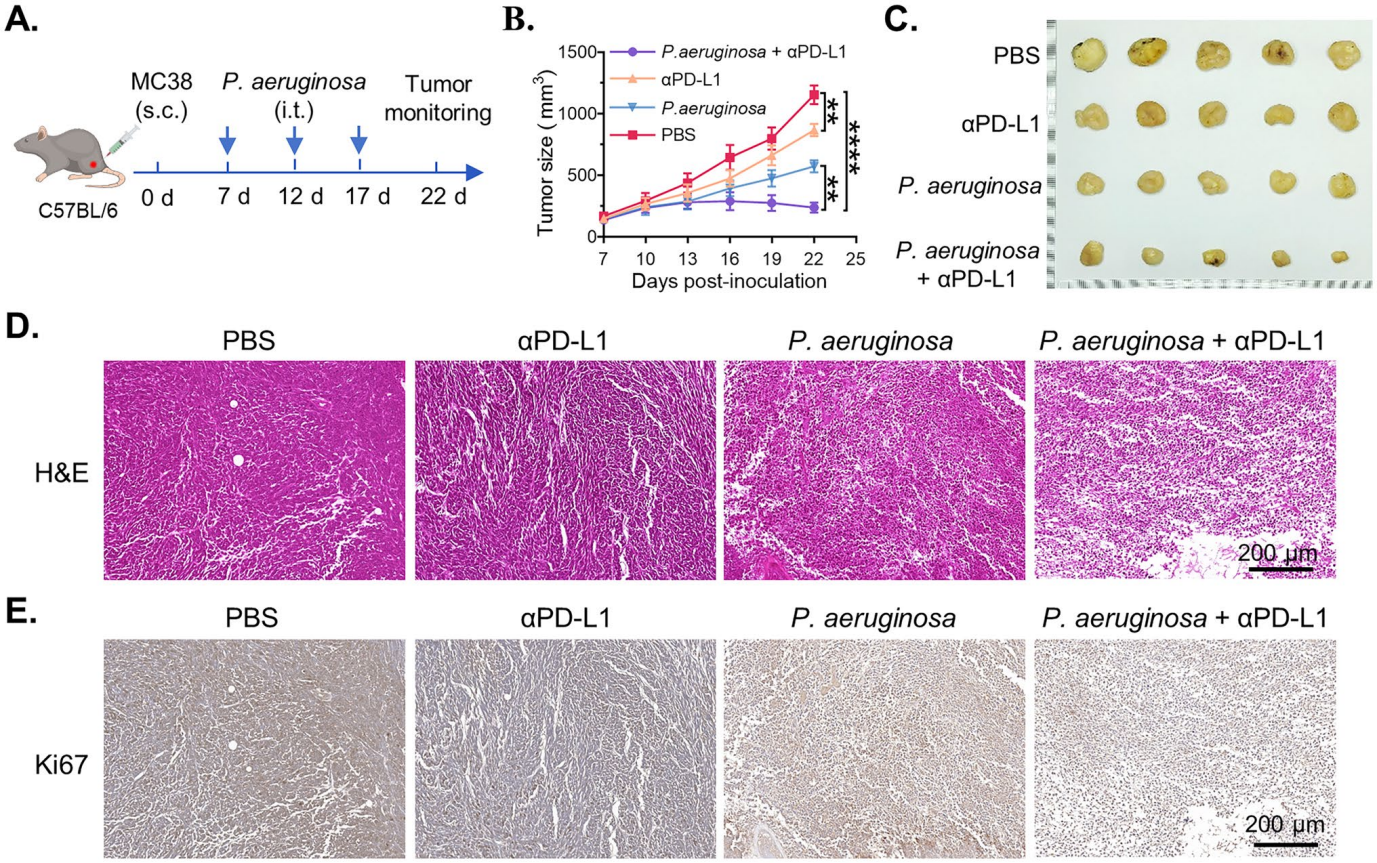
In vivo antitumor activity of *P. aeruginosa* in combination with αPD-L1. **A** The schematic treatment timeline. **B** Tumor growth curves of MC38 tumors  under different treatments. **C** The photograph of tumor tissues at the end of experiments. **D**, **E** Represented photographs of H&E and Ki67 staining of tumor tissues with different treatments. Data are presented as mean ± SD (*n* = 5 mice per group). Statistical significance was determined using one-way ANOVA with Tukey’s post hoc test based on endpoint tumor volumes. * *P* < 0.05, ** *P* < 0.01, *** *P* < 0.001, **** *P* < 0.0001, ns, not significant

To assess the in vivo antitumor efficacy of *P. aeruginosa* and its potential synergy with PD-L1 blockade, MC38 tumor–bearing mice were randomly assigned to four treatment groups (Fig. 6A): PBS control, anti–PD-L1 antibody (αPD-L1) alone, *P. aeruginosa* alone, and combination therapy (*P. aeruginosa* + αPD-L1). Tumor growth was monitored every two days. As shown in the tumor growth curves, both *P. aeruginosa* and anti–PD-L1 monotherapy moderately suppressed tumor progression compared to PBS, whereas the combination group exhibited significantly enhanced tumor growth inhibition throughout the treatment period (Fig. 6B), indicating a synergistic antitumor effect. At the endpoint, tumors were excised and photographed (Fig. 6C). Tumor weights in the combination group were markedly reduced compared to either monotherapy (Fig. S1A), consistent with the growth curve data. Hematoxylin and eosin (H&E) staining of tumor sections revealed more extensive necrotic features in the *P. aeruginosa*- and combination-treated tumors (Fig. 6D), while Ki67 immunohistochemistry showed a significant reduction in proliferative cells in the combination group (Fig. 6E), suggesting enhanced tumor cell killing and proliferation inhibition. In addition, mouse body weights were monitored throughout the study to evaluate treatment tolerability. No significant weight loss was observed in any group (Fig. S1B). Furthermore, histopathological analysis of major organs, including the heart, liver, spleen, lung, and kidney, revealed no severe pathological abnormalities after treatment, and serum biochemical indices (ALT, AST, and BUN) remained within normal ranges across groups (Fig. S3), indicating that both *P. aeruginosa* and αPD-L1, alone or in combination, were well tolerated without inducing systemic toxicity. Collectively, these results demonstrate that intratumoral administration of *P. aeruginosa* exerts potent antitumor activity in vivo and enhances the therapeutic efficacy of immune checkpoint blockade, likely through modulation of the tumor immune microenvironment.

## Discussion

This study provides compelling evidence that *P. aeruginosa* possesses intrinsic antitumor properties against colorectal cancer by inducing tumor cell pyroptosis and remodeling the tumor immune microenvironment, ultimately enhancing the therapeutic efficacy of immune checkpoint blockade. Through a series of in vitro and in vivo experiments, we show that *P. aeruginosa* triggers multiple forms of cell stress and immunogenic cell death, resulting in both direct tumor suppression and enhanced antitumor immune activation.

Mechanistically, *P. aeruginosa* exposure resulted in dose-dependent loss of viability in MC38 cells, accompanied by increased generation of reactive oxygen species (ROS) and membrane rupture. These events culminated in the cleavage of gasdermin E (GSDME), a key executor of pyroptosis in caspase-3–dependent pathways [27–29]. In contrast to apoptosis, pyroptosis is a proinflammatory form of cell death characterized by pore formation in the plasma membrane and release of damage-associated molecular patterns (DAMPs), such as LDH and HMGB1 [30, 31], which were both significantly elevated following *P. aeruginosa* treatment. These findings highlight the ability of *P. aeruginosa* to shift tumor cell fate from non-inflammatory apoptosis toward immunogenic pyroptosis, a phenomenon that may be exploited therapeutically.

Although high concentrations of *P. aeruginosa* resulted in pronounced cytotoxicity in vitro, this effect was accompanied by caspase-3 activation, GSDME cleavage, ROS accumulation, and DAMP release, features that are characteristic of pyroptotic cell death rather than non-specific bacterial overload. Moreover, similar antitumor effects were observed at lower bacterial concentrations, and the in vivo data confirmed that *P. aeruginosa* promoted dendritic cell maturation and CD8^+^ T cell infiltration, supporting its role as a true immunogenic antitumor agent. Importantly, *P. aeruginosa*–induced pyroptosis was accompanied by a marked increase in inflammatory cytokines, including IL-6 and TNF-α, and a significant upregulation of PD-L1 expression on tumor cells. This observation suggests the activation of a tumor-intrinsic feedback loop whereby inflammatory stimuli promote immune evasion through upregulation of immune checkpoint molecules. Notably, the release of DAMPs and cytokines further enhanced the maturation of dendritic cells, as shown by the upregulation of CD80 and CD86 expression on BMDCs exposed to tumor-conditioned media. These results imply that bacterial-induced tumor pyroptosis may serve as an in situ vaccine, enhancing antigen presentation and priming of adaptive immune responses.

Our findings complement prior studies on PA-MSHA, a clinically applied attenuated strain of *P. aeruginosa* that has been shown to inhibit tumor growth and enhance immunotherapy responsiveness through modulation of the tumor immune microenvironment. For instance, PA-MSHA has been reported to suppress EGFR signaling in breast cancer cells and to induce an inflamed tumor phenotype that sensitizes tumors to antiPD-1 therapy [22, 32]. Compared to PA-MSHA, our study highlights that wild-type *P. aeruginosa* not only acts as an immune stimulant but also directly induces GSDME-dependent pyroptosis, thereby generating robust immunogenic signals. This distinction suggests that different *Pseudomonas* strains may exert complementary anti-tumor mechanisms: PA-MSHA primarily through immune regulation and signaling pathway inhibition, and wild-type *P. aeruginosa* through pyroptosis-mediated immunogenic cell death. Future studies integrating these approaches may further optimize bacterial-based strategies for cancer therapy.

In vivo, intratumoral injection of *P. aeruginosa* led to increased expression of TNF-α, IFN-γ, and PD-L1 within tumor tissues, as well as significant maturation of DCs and CD8^+^ T cells. The immune contexture of the tumor was clearly remodeled toward a proinflammatory and immunologically active phenotype. The upregulation of PD-L1 in tumor further highlights the interplay between immune activation and checkpoint engagement. These findings are consistent with the emerging concept that certain microbial stimuli can act as immune adjuvants within tumors, breaking local immune tolerance and converting “cold” tumors into “hot” ones responsive to immunotherapy [18, 33, 34]. Strikingly, the combination of *P. aeruginosa* and anti–PD-L1 therapy resulted in superior tumor suppression compared to either monotherapy. Histopathological analysis revealed extensive necrosis and decreased Ki67 expression in tumors from the combination group, indicating both enhanced cytotoxicity and reduced proliferation. Importantly, no significant body weight loss or apparent toxicity was observed, supporting the safety and feasibility of this therapeutic approach. Further analyses showed that the combination treatment elevated PD-1, LAG3, TIM-3, and CTLA-4 expression in bulk tumor samples, a pattern most consistent with increased T-cell infiltration and activation (Fig. S4A–D). In parallel, IFN-γ and Granzyme B staining confirmed enhanced effector and cytotoxic activity (Fig. S4E–F). Together, these results indicate that the synergy of *P. aeruginosa* with αPD-L1 is likely driven by strengthened immune infiltration and functional reinforcement within the tumor microenvironment.

Our findings align with recent reports that bacteria or their derivatives can function as immune modulators in cancer therapy [35, 36]. While most current strategies rely on engineered bacteria or specific bacterial components, the use of naturally proinflammatory, tumor-restricted bacteria such as* Salmonella* offers a complementary or alternative approach [37–39]. Of note, *P. aeruginosa* has been previously studied for its capacity to activate TME, but its ability to trigger pyroptosis and synergize with checkpoint inhibitors in colorectal cancer has not been well characterized [40, 41]. Our study therefore adds to the growing body of evidence supporting the immunotherapeutic potential of microbial interventions. There are limitations to this study that warrant further investigation. First, the exact upstream signals leading to GSDME cleavage remain to be clarified, including whether *P. aeruginosa* directly activates caspase-3 via secreted toxins or indirectly via stress-induced apoptosis. Second, while our data demonstrate strong local immune activation, systemic antitumor immunity and long-term memory responses were not evaluated. Third, the safety of repeated bacterial administration requires careful consideration, especially in immunocompromised hosts. Future studies should explore the integration of bacterial-based therapy with other immunomodulatory strategies, including STING agonists, adoptive T cell therapy, and gut microbiota modulation.

Another limitation of our study is that the bacteria were not removed after the initial exposure, meaning that tumor cells were continuously cultured in the presence of *P. aeruginosa*. This design was intended to mimic a sustained tumor–bacteria interaction, which may better reflect the tumor microenvironment. Importantly, the culture medium contained penicillin and streptomycin, which effectively restricted bacterial overgrowth, and our preliminary tests confirmed that *P. aeruginosa* did not proliferate extensively under these conditions (Fig. S2). Therefore, the observed inhibitory effects are unlikely to result from uncontrolled bacterial expansion or non-specific cytotoxicity, but primarily reflect cellular responses to bacterial exposure. Nevertheless, we acknowledge that the persistent bacterial presence could still contribute to the observed effects. This represents a shortcoming of the current work, and we have explicitly discussed this limitation to provide a more balanced interpretation of our findings. Together, these results, along with prior knowledge from PA-MSHA, reinforce the concept that microbial interventions can function as immune modulators with multifaceted mechanisms, ranging from immune regulation to direct induction of immunogenic cell death.

## Conclusions

Our findings suggest that *P. aeruginosa* can act as a potent inducer of immunogenic pyroptosis in colorectal cancer, promoting caspase-3-mediated GSDME cleavage, ROS generation, and DAMP release. These events facilitate the remodeling of the tumor immune microenvironment by enhancing dendritic cell maturation, increasing CD8^+^ T cell infiltration, and upregulating PD-L1 expression. Importantly, combination therapy with *P. aeruginosa* and anti-PD-L1 antibody achieves superior tumor control compared to either treatment alone, without detectable systemic toxicity. These results provide preclinical evidence supporting the use of bacteria-mediated pyroptosis as a novel strategy to convert immunologically “cold” tumors into “hot” ones, thereby improving the responsiveness to immune checkpoint blockade in colorectal cancer.

## Supplementary Information

Below is the link to the electronic supplementary material.

Supplementary file1 (DOCX 6688 KB)

## Data Availability

The datasets generated during and/or analysed during the current study are available from the corresponding author on reasonable request.
