# Supplementary material for: Pseudomonas aeruginosa induces tumor pyroptosis and immune activation to enhance checkpoint blockade in colorectal cancer
Source: Cancer Immunol Immunother. 2026 Jan 3;75(1):32. doi: 10.1007/s00262-025-04266-y (PMC12764741; doi:10.1007/s00262-025-04266-y)
Supplement: Supplementary file 1 — Supplementary file1 (DOCX 6688 KB) [file 262_2025_4266_MOESM1_ESM.docx]

**Supplemental Materials**

**
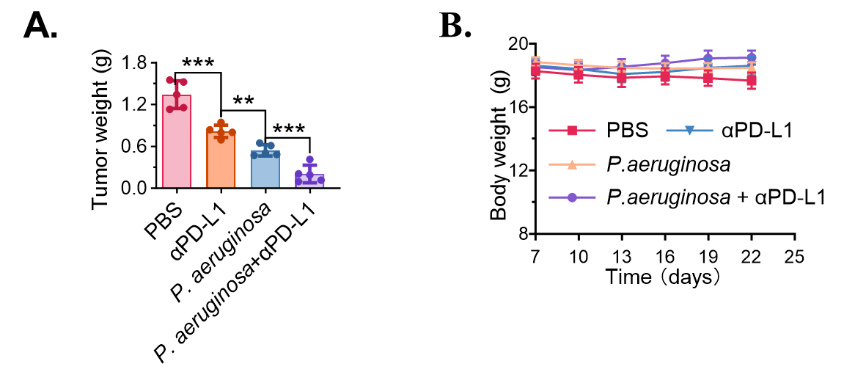
**

**Figure S1**. *In vivo* antitumor activity of *P. aeruginosa* in combination with αPD-L1. **(A)** The tumor weight from mice with different treatments. **(B)** The bodyweight of mice with different treatments. Data are presented as mean ± SD (*n* = 5 mice per group). Statistical significance was determined using one-way ANOVA with Tukey’s post hoc test. *P* < 0.05, ** *P* < 0.01, *** *P* < 0.001.


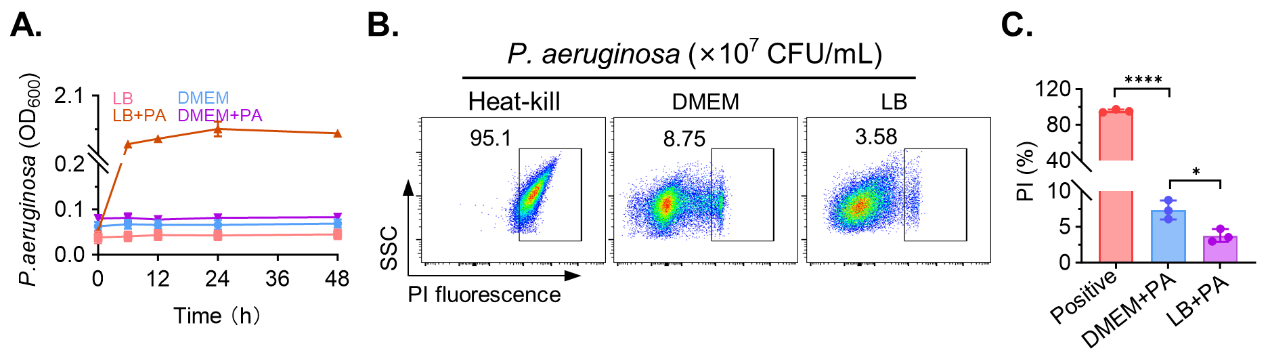


**Figure S2.** **(A)** Growth curves of *P.aeruginosa cultured* in LB medium, DMEM medium, LB medium with *P. aeruginosa* (LB + PA), and DMEM medium with *P. aeruginosa* (DMEM + PA), measured by OD₆₀₀ over time. **(B)** Flow cytometry analysis of *P. aeruginosa* viability by propidium iodide (PI) staining, with representative dot plots showing PI fluorescence intensity. **(C)** Quantification of PI-positive *P. aeruginosa* (%) from flow cytometry data. Data are presented as mean ± SD (*n* = 3 independent biological replicates). Statistical analysis was performed using one-way ANOVA with Tukey’s test. **P* < 0.05; ***P* < 0.01; ****P* < 0.001; *****P* < 0.0001. ns, not significant.


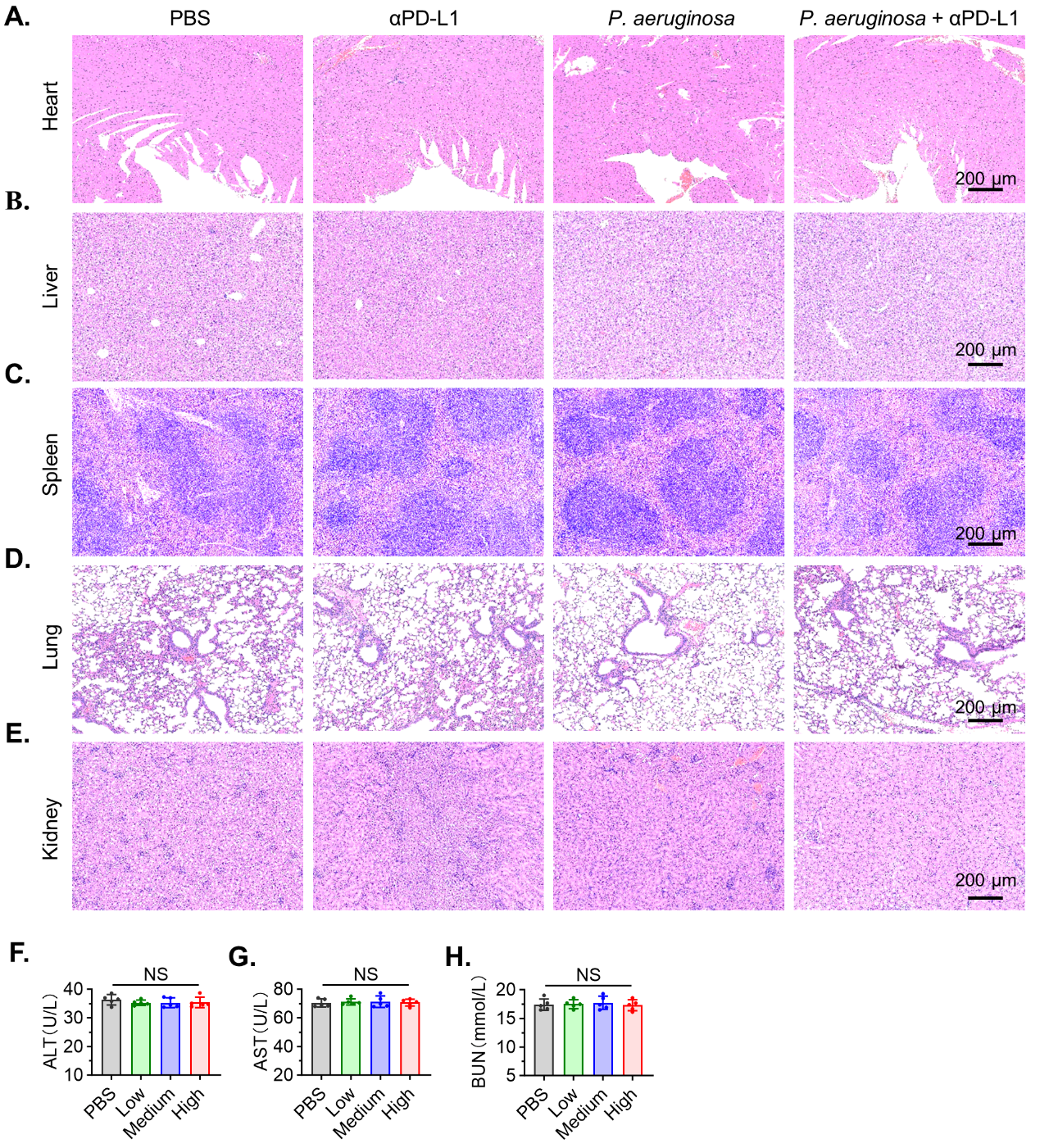
**Figure S3**. **(A-E)** Histopathological analysis of multiple organs following different treatments. Pathological analysis of tissue sections from the heart **(A)**, liver **(B)**, spleen **(C)**, lung **(D)** and kidney **(E)** of mice treated with PBS, *P. aeruginosa* infection, αPD-L1 or *P. aeruginosa* infection combined with αPD-L1. In cardiac tissue, *P. aeruginosa* infection can cause tissue damage, while combined treatment with αPD-L1 does not significantly aggravate the condition. The liver tissue structures of each group were relatively normal. The changes of the spleen tissue combined treatment group were similar to those of the infection group. The tissue showed that the alveolar structure was damaged and the combined treatment did not lead to more serious lesions. No obvious pathological abnormalities were observed in the renal tissues of each group. **(F-H)** Serum biochemical analyses of ALT **(F)**, AST **(G)**, and BUN **(H)** levels revealed no significant differences among groups, supporting the overall biosafety of the treatments. Scale bar = 200 μm. Data are presented as mean ± SD (*n* = 5 mice per group). Statistical significance was determined using one-way ANOVA with Tukey’s post hoc test. ns, not significant. ns, not significant.


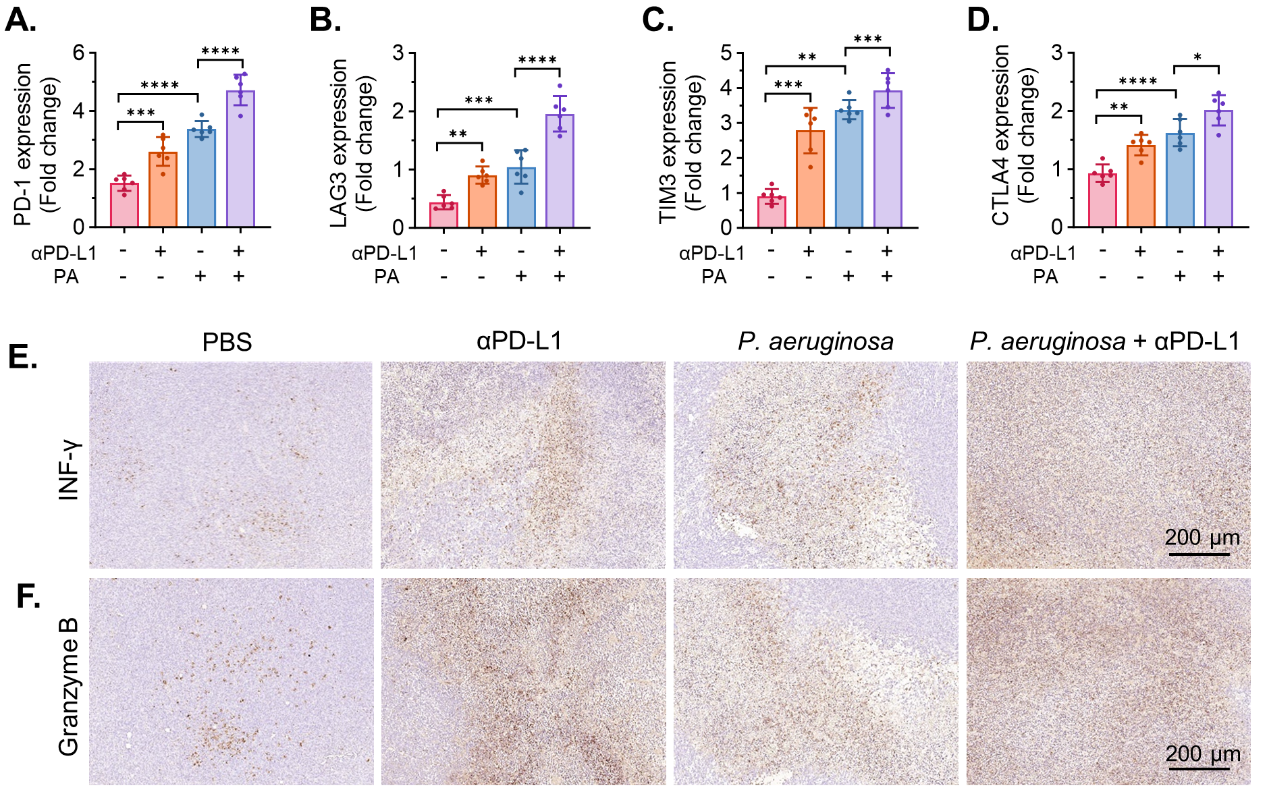


**Figure S4.** **(A-D)** Relative expression levels of of immune checkpoint molecules: PD-1 (**A**), LAG3 (**B**), TIM3 (**C**) and CTLA-4 (**D**) under different treatments (*n* = 6 mice per group). **(E, F)** Representative immunohistochemical staining of immune-related markers in tumor tissues from mice treated with PBS, αPD-L1, *P. aeruginosa*, or *P. aeruginosa* + αPD-L1, including IFN-γ (**E**), and Granzyme B (**F**). Scale bar = 200 μm. Data are presented as mean ± SD. Statistical analysis was performed using one-way ANOVA with Tukey’s test. ns, not significant; *p < 0.05; **p < 0.01; ***p < 0.001; ****p < 0.0001.
